# Self-perceptions of aging mediate the association between illness perception and influenza vaccine hesitancy in older adults with pneumonia during the 2024–2025 influenza season

**DOI:** 10.3389/fpubh.2025.1662035

**Published:** 2025-11-19

**Authors:** Jinyi Liu, Yuting Wang, Zhijuan Zhu, Jing Li, Yating Wang, Shuiqin Xu

**Affiliations:** 1 Department of Nursing, Shaoxing University, Shaoxing, China; 2 Shaoxing Second Hospital, Shaoxing, China

**Keywords:** vaccine hesitancy, seasonal influenza vaccination, self-perceptions of aging, illness perception, pneumonia, mediation analysis

## Abstract

**Background:**

As population aging accelerates, pneumonia cases in older adults continue to rise. Although vaccination effectively prevents influenza and reduces secondary pneumonia risk, hesitancy persists in this demographic. Previous studies have explored the link between illness perception and vaccine hesitancy, but the role of self-perceptions of aging in their relationship remains unclear.

**Methods:**

A cross-sectional study was conducted. From November 2024 to March 2025, 321 older pneumonia inpatients were recruited from a hospital in China. Data on illness perception, self-perceptions of aging, and influenza vaccine hesitancy were collected using the Brief Illness Perception Questionnaire, the brief version of the Attitudes to Aging Questionnaire, and the Influenza Vaccine Hesitancy Scale for individuals aged 60 years and above. Bootstrap sampling (replicates 5,000) was used to examine the mediating role of self-perceptions of aging.

**Results:**

Among participants, 74.1% were unvaccinated. Illness perception positively correlated with influenza vaccine hesitancy (rs = 0.64, *p* < 0.01), while self-perceptions of aging negatively correlated with both (rs = −0.53, −0.75, *p* < 0.01). After controlling for covariates, an indirect association through self-perceptions of aging was observed in the relationship between illness perception and influenza vaccine hesitancy, accounting for 35.36% (95% CI: 22.56–51.39%) of the total association.

**Conclusion:**

This study highlights the importance of self-perceptions of aging toward vaccine take-up among older pneumonia patients. Interventions targeting attitudes toward aging may represent new strategies for increasing influenza vaccination rates.

## 1 Introduction

The World Health Organization estimates that seasonal influenza infects one billion people globally each year and causes 650,000 deaths (1, 2). Approximately 80% of influenza-related excess mortality occurs in people aged 60 years and older (3). Notably, pneumonia incidence among older adults rises with age, representing the most common influenza complication (4). With accelerated population aging, the aged will face a substantial rise in pneumonia cases while remaining highly vulnerable to influenza. Therefore, proactive influenza prevention and management are essential for older patients.

As a preventable disease, influenza can be effectively managed through vaccination. Meta-analyses demonstrate that influenza vaccination decreases hospitalization rates for influenza and pneumonia during epidemic seasons, with efficacy ranging from 25 to 53% (5, 6). Despite these benefits, influenza vaccination rates among older populations fall below the World Health Organization’s recommended 75% coverage (7), e.g., 60% in Canada, 59% in Ireland, and 3.8% in China (8, 9). Influenza vaccine hesitancy has emerged as a prevalent phenomenon, hindering vaccination efforts and compromising herd immunity (10). Defined as a motivational state characterized by reluctance or opposition toward influenza vaccination (11), influenza vaccine hesitancy encompasses hesitancy, refusal, and post-vaccination skepticism, reflecting the complex and dynamic nature of vaccination decision-making.

Illness perception may influence influenza vaccine hesitancy. Illness perception refers to a patient’s cognitive representation of disease progression, shaped by prior knowledge and experience when confronting a health threat (12). A positive disease perception encourages patients to adopt constructive attitudes, engage in healthy behaviors, and achieve favorable health outcomes (13), while a higher negative disease perception leads to elevated hesitation to COVID-19 vaccination (14).

As an important marker of healthy aging, self-perceptions of aging refer to an individual’s comprehensive evaluation of their aging process, encompassing cognitive and emotional experiences and expectations about future aging (15). Self-perceptions of aging tend to become more negative with advancing age, particularly after 65 years (16). Existing research has shown that older adults holding negative attitudes toward aging have lower cognitive abilities, perceive illness as inevitable, and are less likely to utilize preventive healthcare services (17). Furthermore, negative attitudes toward aging may amplify the severity of physical symptoms, leading to decreased engagement in health-promoting behaviors (18). Given that influenza vaccination is a health behavior to prevent influenza and its complications (19), this study proposes that self-perceptions of aging may be associated with influenza vaccine hesitancy.

Healthy cognition has been identified as a predictor of attitudes toward aging (20). Specifically, deteriorating health may amplify negative perceptions of aging, viewing illness as an inevitable consequence of growing older, thereby intensifying fear and resistance toward the aging process (21). This association implies that illness perception may influence attitudes toward aging.

The common-sense model of self-regulation posits that individuals form cognitive and emotional representations of illness when confronting health threats, which then shape their coping strategies and health behaviors (22). Building upon this, the lifespan development model of attitudes to aging emphasizes that individuals’ attitudes toward aging are not static but rather dynamically evolve through healthcare experiences and changes in physical functioning (23). Accordingly, this study proposes that older pneumonia patients, after a health shock, may develop illness perceptions that subsequently alter their attitudes and evaluations of their own aging. Levy’s stereotype embodiment theory further suggests that long-term internalization of negative aging stereotypes may gradually become embodied, influencing physiological, psychological, and behavioral outcomes (24). Such a mechanism implies that negative self-perceptions of aging may reinforce avoidance tendencies toward preventive health behaviors, such as hesitancy toward influenza vaccination. Integrating these theories, this study proposes that disease perception may influence older adults’ decision-making tendencies regarding influenza vaccination by modifying their attitudes toward aging.

Existing research on vaccine hesitancy has focused on external factors, such as vaccine safety, social information, and individual economic capacity, while generally overlooking intrinsic psychological factors related to aging (10). Furthermore, although direct or potential pairwise associations have been identified between illness perception, self-perceptions of aging, and influenza vaccine hesitancy, the role of self-perceptions of aging in mediating the relationship between illness perception and influenza vaccine hesitancy remains unclear.

Drawing upon the theories and findings mentioned above, we hypothesize that disease perception associates influenza vaccine hesitancy both directly and indirectly via self-perceptions of aging. Investigating this relationship will enhance understanding of influenza vaccine hesitancy among vulnerable groups, offering healthcare providers and policymakers a theoretical basis for interventions to improve vaccination rates in this population.

## 2 Methods

### 2.1 Participants

The cross-sectional study using convenience sampling was conducted from November 2024 to March 2025 at a general hospital in China. Inclusion criteria comprised (1) diagnosis of community-acquired pneumonia by a respiratory physician, (2) age ≥60 years (25), and (3) adequate verbal communication skills. Exclusion criteria included (1) allergy to vaccine components or severe influenza vaccine allergy history, (2) psychiatric or cognitive disorders, and (3) severe comorbidities.

### 2.2 Measures

#### 2.2.1 Sociodemographic data and clinical characteristics

A structured questionnaire collected participants’ sociodemographic and clinical data, including age, gender, education level, marital status, residence, monthly income, primary caregiver, smoking and alcohol use histories, chronic conditions, influenza history, pneumonia history, influenza vaccination history, and current vaccination status.

#### 2.2.2 Influenza vaccine hesitation

The Influenza Vaccine Hesitancy Scale for individuals aged 60 years and above was developed by Zhang et al. (26) based on the Influenza Vaccine Hesitancy Scale (VHS-Flu) (27) to assess influenza vaccine hesitancy and its underlying causes in older populations. The scale comprises three dimensions—confidence, risk, and support—with 14 total items. Each item was rated on a 5-point Likert scale (1 = strongly disagree, 5 = strongly agree), with higher scores indicating higher vaccine hesitancy. The original Cronbach’s *α* was 0.85 and 0.85 in this study. See Supplementary material 1 for scale details.

#### 2.2.3 Self-perceptions of aging

The brief version of the Attitudes to Aging Questionnaire (AAQ-BC) developed by Gao et al. (28) evaluates older adults’ attitudes toward aging. It comprises three dimensions: psychosocial loss, physical change, and psychological growth. The 12-item scale was scored on a 5-point Likert scale (1 = completely disagree, 5 = completely agree). Higher scores indicate more positive attitudes toward aging. The Cronbach’s *α* was 0.93 in the original study and 0.73 in this study.

#### 2.2.4 Illness perception

Illness perception was assessed using the Brief Illness Perception Questionnaire (BIPQ) (29), a validated 9-item questionnaire. Item 9 was an open-ended question prompting patients to list their top three perceived causes of illness. The remaining items were scored from 0 to 10 (0 = never, 10 = often), with higher scores reflecting more negative illness perceptions. In this study, Cronbach’s *α* was 0.70.

### 2.3 Procedures

This study adhered to the Declaration of Helsinki and was approved by the Ethics Committee of Shaoxing Second Hospital (Number: 2025025). Eligible patients were invited to participate after being informed of the study’s purpose and their rights. All participants were informed that the survey was anonymous and contained no correct answers. Upon providing written informed consent, participants independently completed the questionnaire. For illiterate subjects, researchers who had undergone standardized training verbally administered the questionnaire and recorded responses. All questionnaires were collected immediately and reviewed for completeness.

### 2.4 Statistical analysis

Statistical analysis was performed using Stata 18.0. Normally distributed data were presented as mean and standard deviation, while non-normally distributed data were expressed as median (25th percentile, 75th percentile) [abbreviated as M (P25, P75)]. The count data were summarized using frequency and percentage. Given the non-normal distribution of illness perception, self-perceptions of aging, and influenza vaccine hesitancy scores, group comparisons used the Mann–Whitney U test or the Kruskal–Wallis test, and correlations were assessed via Spearman correlation. To enhance the sample’s representativeness of the national older adults, we applied post-stratification weights derived from the 2024 China Statistical Yearbook (30). The aging weighting was used in subsequent multiple linear regression and bootstrap mediation analyses. Multiple linear regression identified factors associated with influenza vaccine hesitancy, with robustness tested via ordered probit regression. Bootstrap sampling (5,000 replicates) with weights applied within each resampled regression to estimate the indirect effect of self-perceptions of aging. Model specification was evaluated via the Ramsey RESET test. *p* < 0.05 was considered statistically significant.

## 3 Results

### 3.1 Participants characteristics

The study examined 325 older adults with pneumonia. Following the listwise exclusion of four questionnaires with incomplete data, the final analysis included 321 subjects (95.8%). The majority of participants were aged 60–65 (34.9%), 53.0% were female, and 35.5% had elementary-level education or lower. Most participants were married (91.0%), and over half (56.4%) reported taking care of themselves rather than being dependent on familial or relative care. Regarding lifestyle, 23.1% consumed alcohol, and 14.6% were smokers. Health assessments revealed that 73.2% had 1 to 2 chronic diseases, 79.8% had suffered from influenza, and 12.5% had suffered from pneumonia. Currently, 24.3% of older pneumonia patients report having received influenza vaccination, and 67.0% report knowing vaccinated relatives or neighbors. The majority of participants (90.7%) reported exposure to negative vaccine-related information but lacked professional guidance, with only 3.7% receiving advice from healthcare providers. Weighted participant characteristics are presented in Supplementary material 2.

### 3.2 Factors associated with influenza vaccine hesitancy

As shown in Table 1, significant differences in the score of influenza vaccine hesitancy were observed across groups based on educational level, marital status, primary caregiver, smoking history, chronic diseases, influenza history, pneumonia history, influenza vaccination history, relatives’ and neighbors’ vaccination status, negative news, healthcare advice, and current vaccination status.

**Table 1:** Social-demographic characteristics and comparison of influenza vaccine hesitancy scores in different groups (*N* = 321).

Variable	Group	*N* (%)	Influenza vaccine hesitancy M (P25, P75)	Z/H	*P*
Age ground	60–65	112 (34.9)	40.5 (35.0, 48.0)	4.949	0.293
	66–70	73 (22.7)	41.0 (31.0, 45.0)		
	71–75	61 (19.0)	40.0 (26.5, 47.5)		
	76–80	56 (17.5)	41.0 (26.5, 50.7)		
	>80	19 (5.9)	33.0 (20.0, 48.0)		
Gender	Male	151 (47.0)	42.0 (30.0, 49.0)	−1.715	0.086
	Female	170 (53.0)	39.5 (30.0, 46.0)		
Education level	Illiteracy	67 (20.9)	44.0 (33.0, 50.0)	11.213	0.011
	Primary school and below	114 (35.5)	40.5 (29.7, 48.0)		
	Junior secondary school	96 (29.9)	41.0 (32.0, 47.0)		
	High school/ technical secondary school and above	44 (13.7)	34.5 (23.0, 43.0)		
Marital status	Unmarried/Widowed/Divorced	29 (9.0)	30.0 (23.5, 45.0)	−2.011	0.044
	Married	292 (91.0)	41.0 (31.0, 48.0)		
Residence	Countryside	127 (39.6)	41.0 (29.0, 48.0)	−0.446	0.656
	City	194 (60.4)	40.0 (30.0, 47.0)		
Monthly income	<1, 500 RMB	98 (30.5)	42.5 (28.7, 48.0)	4.220	0.239
	1, 500–3, 500 RMB	113 (35.2)	41.0 (32.0, 49.0)		
	3, 500–6, 000 RMB	97 (30.2)	39.0 (29.5, 46.0)		
	≥6, 000 RMB	13 (4.1)	39.0 (21.5, 44.0)		
Relatives in medical field	Yes	23 (7.2)	45.0 (29.0, 54.0)	−1.477	0.14
	No	298 (92.8)	40.0 (30.0, 48.0)		
Primary caregiver	Spouse	87 (27.1)	43.0 (33.0, 49.0)	10.790	0.029
	Child	40 (12.5)	36.5 (25.7, 47.2)		
	Self	181 (56.4)	40.0 (31.0, 47.0)		
	Relative caregiver	5 (1.5)	33.0 (22.5, 44.0)		
	Non-relative caregiver	8 (2.5)	31.5 (24.0, 40.0)		
Smoking history	Yes	47 (14.6)	45.0 (33.0, 56.0)	6.438	0.040
	Quit smoking	28 (8.7)	39.0 (28.0, 44.5)		
	Never	246 (76.7)	40.0 (29.7, 48.0)		
Drinking alcohol history	Yes	74 (23.1)	45.0 (31.7, 50.0)	5.944	0.051
	Quit drinking	18 (5.6)	41.0 (31.2, 44.2)		
	Never	229 (71.3)	40.0 (29.0, 47.0)		
Chronic disease	0	53 (16.5)	40.0 (34.0, 44.0)	8.040	0.018
	1–2	235 (73.2)	41.0 (30.0, 49.0)		
	≥3	33 (10.3)	30.0 (23.5, 43.5)		
Influenza history	Yes	256 (79.8)	41.5 (31.0, 48.0)	−2.525	0.012
	No	65 (20.2)	37.0 (28.0, 45.0)		
Pneumonia history	Yes	40 (12.5)	35.5 (23.2, 41.7)	−3.399	0.001
	No	281 (87.5)	41.0 (31.0, 48.0)		
Influenza vaccination history	Yes	70 (21.8)	25.5 (20.7, 30.0)	−9.756	<0.001
	No	251 (78.2)	44.0 (37.0, 49.0)		
Relatives’ and neighbors’ vaccination status	I do not know	106 (33.0)	44.0 (35.0, 49.0)	63.072	<0.001
	Relatives vaccinated	122 (38.0)	43.0 (37.0, 49.0)		
	Neighbors vaccinated	15 (4.7)	39.0 (32.0, 45.0)		
	Both vaccinated	78 (24.3)	28.0 (21.0, 37.2)		
Negative news	Yes	291 (90.7)	41.0 (31.0, 48.0)	−3.406	0.001
	No	30 (9.3)	28.5 (24.0, 38.7)		
Healthcare advice	Yes	12 (3.7)	27.0 (23.0, 35.7)	−2.967	0.003
	No	309 (96.3)	41.0 (30.0, 48.0)		
Current vaccination status	Yes	78 (24.3)	25.0 (21.0, 29.0)	133.387	<0.001
	Unvaccinated but planning to	5 (1.6)	23.0 (15.5, 27.5)		
	No	238 (74.1)	44.0 (38.0, 49.0)		

Data are presented as frequency (%) and median (25th percentiles, 75th percentiles); *p*-values were calculated with Mann–Whitney U or Kruskal-Wallis tests.

### 3.3 Correlation analysis of illness perception, self-perceptions of aging, and influenza vaccine hesitancy

Pearson correlation analysis showed a positive correlation between illness perception and influenza vaccine hesitancy (rs = 0.64, *p* < 0.01) and a negative correlation between self-perceptions of aging and influenza vaccine hesitancy (rs = −0.75, *p* < 0.01). In addition, illness perception and self-perceptions of aging were also inversely correlated (rs = −0.53, *p* < 0.01, Table 2).

**Table 2:** Descriptive statistics and correlation analysis of illness perception, self-perceptions of aging and influenza vaccine hesitancy (*N* = 321).

Variable	M (P25, P75)	Illness perception	Self-perceptions of aging	Influenza vaccine hesitancy
Illness perception	5.50 (4.50,6.13)	1	—	—
Self-perceptions of aging	3.00 (2.33,3.33)	−0.53**	1	—
Influenza vaccine hesitancy	2.85 (2.14,3.42)	0.64**	−0.75**	1

— Indicates duplicate data; **P < 0.01.

### 3.4 Mediation analysis estimating indirect associations between illness perception and influenza vaccine hesitancy through self-perceptions of aging

**Figure 1:**
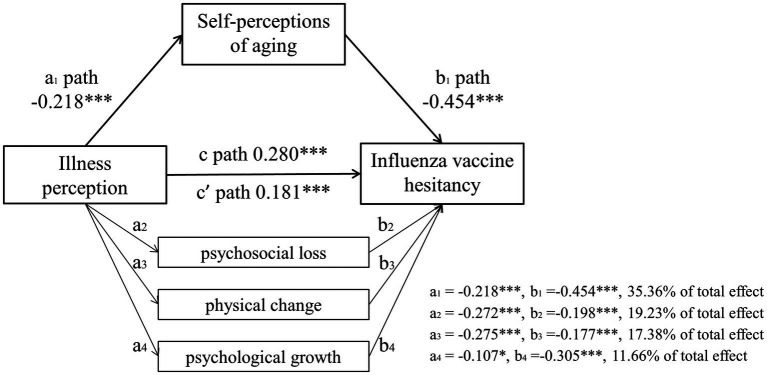
Path diagram of the mediation analysis estimating indirect associations between illness perception and influenza vaccine hesitancy through self-perceptions of aging and its subscales (psychosocial loss, physical change, psychological growth). a1/2/3/4 path: the effect of illness perception on self-perceptions of aging, psychosocial loss, physical change, and psychological growth; b1/2 /3/4 path: the effect of self-perceptions of aging, psychosocial loss, physical change, and psychological growth on influenza vaccine hesitancy; c path: the total effect of illness on influenza vaccine hesitancy; c’ path: the direct effect of illness perception on influenza vaccine hesitancy. *p < 0.05; **p < 0.01;***p < 0.001.

After controlling for education level, marital status, primary caregiver, smoking history, chronic diseases, influenza history, pneumonia history, influenza vaccination history, relatives’ and neighbors’ vaccination status, negative news, healthcare advice, and current vaccination status, we investigated the indirect role of self-perceptions of aging in the relationship between illness perception and influenza vaccine hesitancy. Results showed that illness perception positively predicted influenza vaccine hesitancy (*β* = 0.280, *t* = 6.58, *p* < 0.001), remaining significant after controlling for self-perceptions of aging (*β* = 0.181, *t* = 4.71, *p* < 0.001). It also negatively predicted self-perceptions of aging (*β* = −0.218, *t* = −5.03, *p* < 0.001). Robustness tests using ordered probit regression yielded consistent directional results (*p* < 0.001) (Figure 1; Table 3). The Ramsey RESET test indicated no significant specification error in Model 2, incorporating the mediating variable of self-perceptions of aging (*F* = 0.72, *p* = 0.396) (Table 4). Unweighted multiple linear regression results are presented in Supplementary material 3.

**Table 3:** Mediation analysis estimating indirect associations between illness perception and influenza vaccine hesitancy via self-perceptions of aging (*N* = 321).

Model	Model 1			Model 2			Model 3		
Dependent variable	Influenza vaccine hesitancy			Self-perceptions of aging			Influenza vaccine hesitancy		
Indicators	Weighted multiple linear regression		Robustness check, Coef. (z)	Weighted multiple linear regression		Robustness check, Coef. (z)	Weighted multiple linear regression		Robustness check, Coef. (z)
	Coef. (t)	Robust Std. Err.		Coef. (t)	Robust Std. Err.		Coef. (t)	Robust Std. Err.	
Illness perception	0.280 *** (6.71)	0.042	0.588 *** (5.74)	−0.218 *** (−5.13)	0.043	−0.436 *** (−4.35)	0.181 *** (4.81)	0.038	0.439 *** (4.63)
Self-perceptions of aging							−0.454 *** (−7.36)	0.063	−1.093 *** (−6.24)
R^2^	0.651			0.458			0.728		
Pseudo R^2^			0.129			0.088			0.163
F	48.19***		18.72***	24.96***		12.95***	69.85***		23.36***

Robustness checks were conducted using ordered probit regression with probability weights. Model 1: examines the total effect of illness perception on influenza vaccine hesitancy (c path). Model 2: examines the effect of illness perception on self-perceptions of aging (a1 path). Model 3: examines the direct effect of illness perception on vaccine hesitancy after controlling for self-perceptions of aging (c’ path). ***P < 0.001.

**Table 4:** Model specification bias test (*N* = 321).

Model	Dependent variable	Predictors	ŷ^2^ Coefficient	SE	F	P
Model 1	Influenza vaccine hesitancy	Illness perception + controls	0.278	0.076	13.8	< 0.001
Model 2	Influenza vaccine hesitancy	Illness perception + self-perceptions of aging + controls	0.044	0.051	0.72	0.396

The Ramsey reset test (adding a squared fit term) was used for model specification testing. SE: Standard error.

Bootstrap 95% CIs for direct and indirect effects excluded zero (Table 5). Furthermore, the direct effect of illness perception on influenza vaccine hesitancy remained significant after introducing the mediating variable of self-perceptions of aging, supporting partial mediation. The total effect size was 0.280, and the direct effect size was 0.181. Thus, self-perceptions of aging mediated 35.36% (95% CI: 22.56–51.39%) of the total effect of illness perception on influenza vaccine hesitancy. The model depicting the indirect association is shown in Figure 1. Moreover, significant indirect associations via all three dimensions of self-perceptions of aging were observed (Figure 1; Supplementary material 4).

**Table 5:** Direct and indirect effects of illness perception on influenza vaccine hesitancy (*N* = 321).

Effect type	Coef.	SE	95% CI	Std. Coef.	Proportion of effect (95% CI)
Total effect (c path)	0.280	0.043	0.198–0.371	0.361	
Direct effect (c’ path)	0.181	0.039	0.107–0.265	0.234	64.64% (46.98–76.60%)
Indirect effect (a1*b1)	0.099	0.021	0.062–0.146	0.127	35.36% (22.56–51.39%)

Coef., unstandardized coefficient; Std. Coef., completely standardized coefficient; SE, standard error; 95% CI, the 95% confidence intervals.

## 4 Discussion

This study identified a significant indirect association of self-perceptions of aging in the relationship between illness perception and influenza vaccine hesitancy. Our findings indicate that higher illness perception was significantly associated with greater influenza vaccination hesitancy. Self-perceptions of aging accounted for 35.36% (95% CI: 22.56–51.39%) of this relationship.

The current influenza vaccination status among older pneumonia patients in our study (24.3%) was higher than that reported by Fan et al. (9) for the general older population (3.8%). This difference is consistent with the potential impact of the free vaccination policy implemented in September 2024. Financial constraints are a well-documented barrier to vaccination uptake (10). In addition, our observation is consistent with the notion that individuals prioritize preventive behaviors that are directly related to their specific health problems (19, 31). Despite this improvement, the current influenza vaccination status remains substantially lower than rates reported in other countries, including South Korea (75.8%), the United States (71.5%), Australia (70.9%), the United Kingdom (70.8%), and New Zealand (68%) (8). This disparity could be explained by deeply rooted health beliefs in Chinese culture, particularly among older adults, who often favor “natural immunity” (32). In collectivist cultures, healthcare decisions are shaped not only by individual preference but also by familial and societal expectations (33). For example, traditional filial piety emphasizes children’s responsibility in safeguarding the health of their older family members (34). This could lead older adults to partially delegate decision-making authority—such as vaccine choices—to their children, adopting a passive stance toward such matters themselves.

This study found that education level, marital status, chronic disease history, caregiver status, smoking history, prior influenza or pneumonia infection, and influenza vaccination history significantly influenced influenza vaccination hesitancy, aligning with previous studies (10, 35–37). In addition, information channels exert a significant influence on influenza vaccination hesitancy (38). Consistent with previous research (39), influenza vaccination hesitancy is significantly influenced by family and friends’ vaccination behaviors and professional healthcare recommendations. As an open system, the common-sense model of self-regulation acknowledges that external information shapes internal representations. Healthcare providers, as reliable information sources, can shape patients’ positive disease representations and coping assessments through their attitudes (40). However, reliance on unofficial channels disseminating negative information erodes trust in the healthcare system, fosters negative disease representations, and exacerbates vaccine hesitancy (41). We highlight that exposure to negative vaccine information significantly increased influenza vaccine hesitancy, not only from social networks (42). These findings underscore the need for targeted interventions among high-risk groups, such as those with limited education, inadequate social support, and restricted information access.

The common-sense model of self-regulation posits that individuals form disease representations in response to health threats. This study suggests that such representations are directly associated with the evaluation of coping behaviors. Specifically, stronger disease perception correlates with higher vaccine hesitancy—a relationship also reported for COVID-19 vaccine research (14). This could stem from insufficient health literacy among those with heightened disease perception, leading to excessive focus on vaccine adverse effects and weakened adherence to health behaviors (43). The mediation analysis indicates that disease perception was not only directly associated with influenza vaccine hesitancy but also indirectly associated with it through self-perceptions of aging. Notably, all three subdimensions of self-perceptions of aging—psychosocial loss, physical changes, and psychological growth—served as significant components of this indirect pathway. Psychosocial loss contributed most substantially, implying that fear of social devaluation constitutes an important psychological barrier to healthy behaviors. When hospitalized with pneumonia in old age, the illness experience is not merely a physiological shock but may activate or reinforce negative aging stereotypes that equate aging with diminished social value, thereby shaping negative self-perceptions of aging (44). Negative attitudes toward aging contribute to fatalistic beliefs (e.g., “aging inevitably worsens health”), a cognitive bias that fosters vaccine hesitancy by undervaluing benefits while overestimating risks (45).

Moreover, unlike chronic disease management, acute pneumonia hospitalization forces older patients to directly and intensely confront functional decline and physical vulnerability. This experience may be internalized as accelerated aging. Previous research indicates that a single acute illness can trigger or exacerbate frailty in older adults. Hospitalized pneumonia patients are likely undergoing this process (46). They may attribute functional limitations such as dyspnea and fatigue caused by pneumonia to irreversible aging, thereby reinforcing the negative stereotype that “aging equals decline.”

The common-sense model of self-regulation suggests that individuals’ health behavior decisions constitute a continuous and dynamic feedback process. Considering the identified indirect association via self-perceptions of aging, clinical interventions could reshape older adults’ attitudes toward aging. Integrating educational modules that address negative aging stereotypes into rehabilitation programs is recommended. These modules should clarify to patients that physical function can be improved through exercise, nutrition, and other means even in advanced age. This helps reshape their belief in health control and dismantles the negative perception that “disease equals irreversible aging” (47). Furthermore, active participation in rehabilitation training aimed at restoring independent living abilities should be encouraged. Even small improvements should be positively reinforced and tracked visually via journals, helping patients view recovery as a sign of health rather than aging failure (48). Organizing intergenerational activities with schools or volunteer groups can also be beneficial. In such settings, older adults in recovery can share traditional skills or life wisdom with younger generations, thereby enhancing self-worth and fostering positive attitudes toward aging (49).

This study has several limitations. First, as a single-center study conducted at a general hospital in eastern China using convenience sampling, the findings may have limited generalizability and could involve selection bias, particularly by excluding individuals without access to healthcare services. Second, although critically ill and cognitively impaired patients were excluded, no standardized frailty or cognitive assessments were used—factors that may affect attitudes toward aging and medical decisions. Thus, applying these results to older populations with frailty or cognitive impairment requires caution. The reliability of measurement tools was also borderline, potentially leading to conservative estimates of observed indirect associations. Third, self-reported data are subject to social desirability and recall biases, and the reading of questions to illiterate participants by researchers may have introduced bias. Finally, despite low rates of missing data unlikely to affect results, listwise deletion was used. Future research could consider more advanced methods for handling missing data. To improve reliability and generalizability, subsequent research should adopt multi-center stratified sampling methods, employ standardized tools with high reliability, and incorporate objective data.

## 5 Conclusion

This study highlights the risk factors for influenza vaccine hesitancy among older Chinese patients with pneumonia, demonstrating that illness perception and self-perceptions of aging are critical cognitive and psychological determinants. It was found that self-perceptions of aging were indirectly associated with the relationship between illness perception and influenza vaccine hesitancy, accounting for 35.48% of the effect. These findings offer new evidence regarding the psychological mechanisms of influenza vaccine hesitancy. We recommend integrating disease education and self-perceptions of aging assessments into pneumonia care, and providing targeted psychological support may further improve vaccination uptake.

## Data Availability

The original contributions presented in the study are included in the article/Supplementary material, further inquiries can be directed to the corresponding author/s.
