# Supplementary material for: Self-perceptions of aging mediate the association between illness perception and influenza vaccine hesitancy in older adults with pneumonia during the 2024–2025 influenza season
Source: Front Public Health. 2025 Nov 19;13:1662035. doi: 10.3389/fpubh.2025.1662035 (PMC12672264; doi:10.3389/fpubh.2025.1662035)
Supplement: Supplementary file 1 [file Data_Sheet_1.docx]

Supplementary Material

**Supplementary Material 1.** Influenza vaccine hesitancy scale for people aged 60 years and over

| Dimensions | Entries |
| --- | --- |
| Confidence | 1 Getting the influenza vaccine is important for my health. |
|  | 2 Getting the influenza vaccine is a good way to protect me from the influenza and its complications. |
|  | 3 The influenza vaccine is effective. |
|  | 4 Getting the influenza vaccine is also important for the health of those around me. |
|  | 5 The influenza vaccine provided by healthcare institutions is beneficial to me. |
|  | 6 The information I have learned about the influenza vaccine is reliable and trustworthy. |
|  | 7 About getting the influenza vaccine, I follow the advice of healthcare professionals. |
| Risk | 1 I am worried that getting the influenza vaccine might give me the flu. |
|  | 2 I am worried that getting the influenza vaccine might cause serious side effects. |
|  | 3 I am worried that getting the influenza vaccine might affect my long-term health. |
|  | 4 I believe my risk of catching influenza is low. |
| Support | 1 My family supports me in getting the influenza vaccine. |
|  | 2 I can afford the cost of getting the influenza vaccine. |
|  | 3 It is convenient for me to go to a vaccination center to get the influenza vaccine. |

**Supplementary Material 2.** Comparison of characteristics distribution before and after age weighting (N=321)

| **Variable** |  | **Proportion,% (unweighted)** | **Proportion,% (weighted)** |
| --- | --- | --- | --- |
| Age ground | 60-65 | 34.9 | 27.4 |
|  | 66-70 | 22.7 | 26.4 |
|  | 71-75 | 19.0 | 20.9 |
|  | 76-80 | 17.5 | 12.8 |
|  | >80 | 5.9 | 12.5 |
| Gender | Male | 47.0 | 48.2 |
|  | Female | 53.0 | 51.8 |
| Education level | Illiteracy | 20.9 | 20.8 |
|  | Primary school and below | 35.5 | 36.4 |
|  | Junior secondary school | 29.9 | 28.9 |
|  | High school/ technical secondary school and above | 13.7 | 13.9 |
| Marital status | Unmarried/ Widowed/ Divorced | 9.0 | 9.2 |
|  | Married | 91.0 | 90.8 |
| Residence | Countryside | 39.6 | 40.3 |
|  | City | 60.4 | 59.7 |
| Monthly income | <1500 RMB | 30.5 | 31.8 |
|  | 1500-3500 RMB | 35.2 | 34.7 |
|  | 3500-6000 RMB | 30.2 | 29.5 |
|  | ≥6000 RMB | 4.1 | 4.0 |
| Relatives in medical field | Yes | 7.2 | 6.7 |
|  | No | 92.8 | 93.3 |
| Primary caregiver | Spouse | 27.1 | 26.7 |
|  | Child | 12.5 | 14.7 |
|  | Self | 56.4 | 54.2 |
|  | Relative caregiver | 1.5 | 1.4 |
|  | Non-Relative caregiver | 2.5 | 3.0 |
| Smoking history | Yes | 14.6 | 14.1 |
|  | Quit smoking | 8.7 | 8.0 |
|  | Never | 76.7 | 77.9 |
| Drinking alcohol history | Yes | 23.1 | 22.4 |
|  | Quit drinking | 5.6 | 5.4 |
|  | Never | 71.3 | 72.2 |
| Chronic disease | 0 | 16.5 | 15.2 |
|  | 1-2 | 73.2 | 74.2 |
|  | ≥3 | 10.3 | 10.6 |
| Influenza history | Yes | 79.8 | 80.5 |
|  | No | 20.3 | 19.5 |
| Pneumonia history | Yes | 12.5 | 14.3 |
|  | No | 87.5 | 85.7 |
| Influenza vaccination history | Yes | 21.8 | 23.8 |
|  | No | 78.2 | 76.2 |
| Relatives’ and neighbors’ vaccination status | I don't know | 33.0 | 33.2 |
|  | Relatives vaccinated | 38.0 | 35.4 |
|  | Neighbors vaccinated | 4.7 | 4.2 |
|  | Both vaccinated | 24.3 | 27.2 |
| Negative news | Yes | 90.7 | 91.4 |
|  | No | 9.3 | 8.6 |
| Healthcare advice | Yes | 3.7 | 3.5 |
|  | No | 96.3 | 96.5 |
| Current vaccination status | Yes | 24.3 | 21.1 |
|  | Unvaccinated but planning to | 1.6 | 1.4 |
|  | No | 74.1 | 73.5 |

**Supplementary Material 3.** Mediation analysis estimating indirect associations between illness perception and influenza vaccine hesitancy via self-perceptions of aging (N=321)

| **Model** | **Model 1** | | | **Model 2** | | | **Model 3** | | |
| --- | --- | --- | --- | --- | --- | --- | --- | --- | --- |
| **Dependent variable** | **Influenza vaccine hesitancy** | | | **Self-perceptions of aging** | | | **Influenza vaccine hesitancy** | | |
| **Indicators** | **Multiple Linear Regression, β(t)** | | **Robustness Check, Coef. (z)** | **Multiple Linear Regression, β(t)** | | **Robustness Check, Coef. (z)** | **Multiple Linear Regression, β(t)** | | **Robustness Check, Coef. (z)** |
|  | **Unweighted** | **Weighted** |  | **Unweighted** | **Weighted** |  | **Unweighted** | **Weighted** |  |
| Illness perception | 0.312  ***  (10.28) | 0.280  ***  (6.71) | 0.588  ***  (5.74) | -0.253  ***  (-7.91) | -0.218  ***  (-5.13) | -0.436  ***  (-4.35) | 0.202  ***  (6.82) | 0.181  ***  (4.81) | 0.439  ***  (4.63) |
| Self-perceptions of aging |  |  |  |  |  |  | -0.434  ***  (-9.02) | -0.454  ***  (-7.36) | -1.093  ***  (-6.24) |
| R^2 | 0.656 | 0.651 |  | 0.465 | 0.458 |  | 0.729 | 0.728 |  |
| Pseudo R² |  |  | 0.129 |  |  | 0.088 |  |  | 0.163 |
| F | 45.13*** | 48.19*** | 18.72*** | 20.50*** | 24.96*** | 12.95*** | 58.68*** | 69.85*** | 23.36*** |

Note. Probability-weighted and unweighted multiple linear regression results were reported. Robustness checks were conducted using ordered probit regression with probability weights. Model 1: examines the total effect of illness perception on influenza vaccine hesitancy (c path). Model 2: examines the effect of illness perception on self-perceptions of aging (a1 path). Model 3: examines the direct effect of illness perception on vaccine hesitancy after controlling for self-perceptions of aging (c' path). *** P < 0.001.

**Supplementary Material 4.** The indirect effects of self-perceptions of aging and its subscales between illness perception and influenza vaccine hesitancy (N=321)

| Mediator Variable | a path | b path | Indirect effect | 95% CI | Proportion of total effect (95% CI) |
| --- | --- | --- | --- | --- | --- |
| Self-perceptions of aging | -0.218*** | -0.454*** | 0.099 | 0.062 - 0.146 | 35.36% (23.40% - 53.02%) |
| psychosocial loss | -0.272*** | -0.198*** | 0.054 | 0.031 – 0.087 | 19.23% (10.82 – 32.43%) |
| physical change | -0.275*** | -0.177*** | 0.049 | 0.026 - 0.080 | 17.38% (9.25% - 30.22%) |
| psychological growth | -0.107* | -0.305*** | 0.033 | 0.004 - 0.067 | 11.66% (1.65% - 24.21%) |

Note. 95% CI: the 95% confidence intervals. * P < 0.05; ** P < 0.01;*** P < 0.001.
